# Parosteal osteosarcoma mimicking osteochondroma: A radio-histologic approach on two cases

**DOI:** 10.1186/2045-3329-1-2

**Published:** 2011-07-25

**Authors:** Zafiria G Papathanassiou, Marco Alberghini, Philippe Thiesse, Marco Gambarotti, Giuseppe Bianchi, Cristina Tranfaglia, Daniel Vanel

**Affiliations:** 1 The Rizzoli Orthopedic Institute, Research Department, Bologna, Italy; 2 The Rizzoli Orthopedic Institute, Pathology Department, Bologna, Italy; 3 Centre Léon Bérard, Radiology, Lyon, France; 4 The Rizzoli Orthopedic Institute, Orthopedic Surgery Department, Bologna, Italy; 5 San Orsola Hospital, Nuclear Medicine, Bologna, Italy

## Abstract

**Objective:**

Parosteal osteosarcoma is a well-differentiated variant of osteosarcoma that affects the surface of the bone. The imaging pattern is very typical. We report two cases mimicking an osteochondroma, radiologically and histologically and propose an explanation.

**Material:**

The review of 86 parosteal osteosarcomas of bone revealed this atypical pattern only once. A consultation case was received in the same time, and added to ours. Patients were 28 years old and 56 years old females. Imaging studies included two radiographs, two CTscans, one MRI examination and one bone scan and the results were compared to histology.

**Results:**

On imaging, both lesions presented as ossified lobulated masses attached with a broad base to the underlying cortex. No radiolucent cleft separated the masses and the host bone and cortex continuity between the mass and the femur was seen, with medullary communication. The marrow of the mass had a different density and intensity compared to normal marrow. So, there were features of an osteochondroma (cortex and medullary continuity) and of a parosteal osteosarcoma (ossified marrow). Pathological assessment on the final specimen confirmed the presence of low-grade parosteal osteosarcomas, after an erroneous diagnosis of osteochondroma on the initial biopsy.

**Conclusions:**

Parosteal osteosarcoma can be rarely confused with osteochondroma. A radiologic-pathologic correlation is essential. Cortex continuity is the most misleading imaging feature that may occur in parosteal osteosarcomas. A knowledge of this misleading pattern will help diagnose the lesion from the beginning.

## Introduction

Parosteal osteosarcoma (POS) is a slow-growing tumor which originates from the outer layer of the periosteum and represents 65% of surface osteosarcomas [1] and in our database accounts approximately for 4, 8% of all osteosarcomas [2]. Unlike conventional osteosarcomas, it involves an older age group typically in the 3^rd ^and 4^th ^decades of life and shows a slight female predilection [1,3,4]. The most common location of a POS is the posterior aspect of the distal femur accounting for approximately two thirds of all cases [5]. Confusion may rarely occur in differentiating POS from the sessile variant of osteochondroma on imaging studies. Herein, we report two cases of POS with sessile configurations arising from the posterior distal femur that simulated osteochondromas radiologically and histologically on the initial biopsy and emphasize on the imaging features that may lead to an erroneous diagnosis.

## Materials and methods

The study included two female patients aged 56 and 24 years old respectively. Both patients referred to our institution for evaluation of a palpable mass located at the postero-lateral aspect of the distal femur. Information regarding clinical history, physical examination and laboratory tests was recorded. Retrospective evaluation of the available imaging studies was carried out. Both cases were assessed with radiographs (antero-posterior and lateral views) and CT scans before and after contrast medium i.v. infusion. Additionally, one patient underwent a bone scan and an MRI study which included T1WSE before and after gadolinium administration and T2W FAT SAT sequences. Imaging results were compared with the initial biopsy findings and final histopathologic diagnosis.

## Results

Both patients presented with focal swelling and mechanical blockage of the left knee. The duration of symptoms was 24 and 36 months for each case with no related trauma or infection during this period. Physical examination revealed two hard, immobile tender masses at the postero-lateral aspect of the distal left femur associated with flexion restriction of the joint at 90° degrees. There was no vascular or neurological deficit of the affected limp. The patients did not notice any significant enlargement of the swelling during the period of the symptoms. Routine blood tests were not significant for any pathological findings.

**Figure 1:**
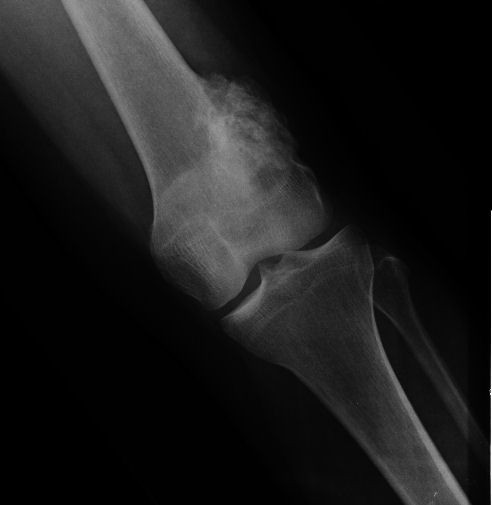
**Case 1 - AP view**.

**Figure 2:**
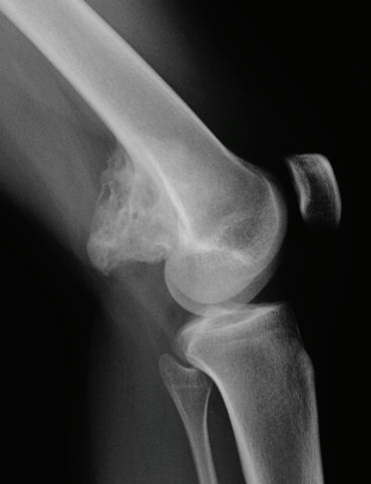
**Case 1 - lateral view**. Radiographs demonstrate a sessile ossified mass arising from the posterior surface of the left distal femur metaphysis. No cleavage plane is observed.

**Figure 3:**
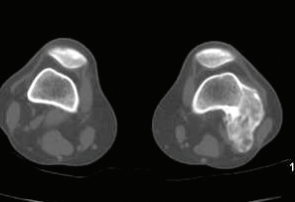
**Case 1 - On axial CT scan, the tumor is continuous within host bone**. Nonmineralized areas are located peripherally.

**Figure 4:**
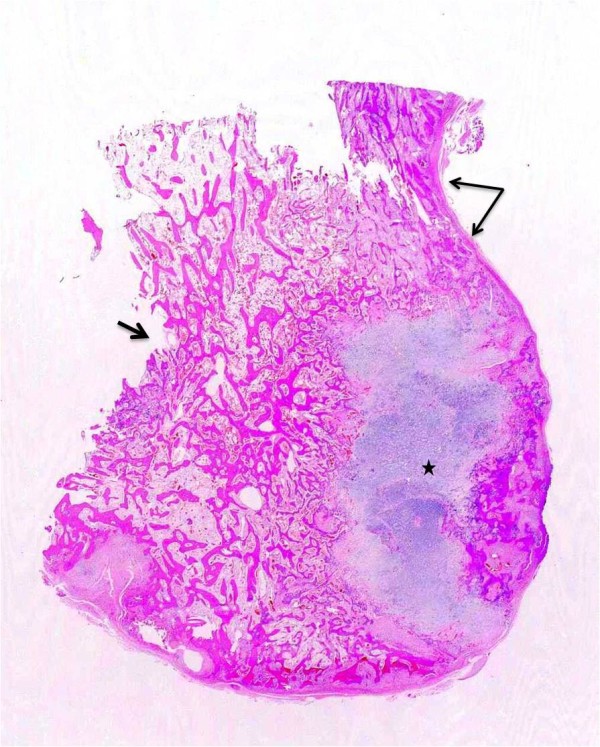
**Case 1 - On the gross specimen continuity of the cortex (long arrows), disappearance of the normal cortex (short arrow), and peripheral cartilage (asterisk) are clear**. Anastomosing trabecular-appearing bone is surrounded by a moderately cellular spindle cell proliferation.

**Figure 5:**
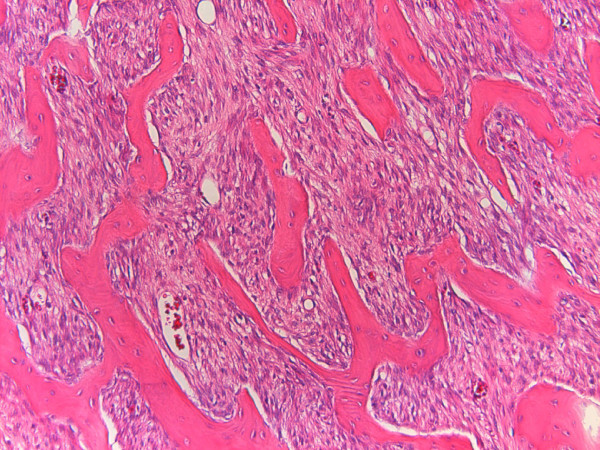
**Case 1 - Spindle cells show mild atypia (Broders grade 2) (Hematoxiline&Eosine, 10 × magnification)**.

Radiographs showed two "exophytic" mineralized masses with a sessile configuration attached to the posterior surface of the left distal femur metaphysis (case1: Figures 1, 2, 3, 4, 5, &6, case 2: Figures 7, 8, 9, 10, &11; radiographs: 1-2, 7-8). No radiolucent cleft separated the masses from the host bone. The lesions maximum diameters were 5, 5 cm and 6 cm. Computed tomography demonstrated an intact and continuous femoral cortex encircling the masses. No definite separation between the medullar portion of the masses and the femur could be ascertained and they seemed to communicate. Moreover, one lesion (5, 5 cm) had mild involvement of the medullary canal on CT scan. Small unmineralized areas were observed mainly at the periphery of the lesions (Figures 3, &9). On MR T1W images, one mass (5, 5 cm) had predominantly low SI compared to adjacent muscles and exhibited heterogeneous enhancement post gadolinium administration (Figures 10, &11). T2W images with fat saturation displayed a heterogeneous lesion containing areas of low to intermediate SI mixed with hyperintense areas, located peripherally. A coexisting popliteal cyst was also noticed at the medial aspect of the popliteal fossa. T2 FAT SAT images additionally documented limited invasion of the medullar canal by the tumor; a finding that was initially suggested on CT scan (Figure 12). The mass was also hot on bone scintigraphy. Both radiographs and CT scans favored the diagnosis of an osteochondroma without excluding the possibility of a low-grade malignancy, like POS. A low-grade peripheral chondrosarcoma was not considered, the tumor developing inside the marrow. Initial biopsy results for both lesions suggested an osteochondroma. A subsequent surgical biopsy that was performed on one of the masses (5, 5 cm) yielded results suggestive of a low grade POS with a cartilaginous component. Owing to cortex continuity, the differential diagnosis was narrowed between a sessile osteochondroma and a low-grade POS and finally complete marginal resections were performed on both cases. Histologic evaluation of the resected specimens confirmed parosteal osteosarcomas (grade II) containing cartilaginous components (Figure 4, 5, &6). For one of the cases (5, 5 cm), because it was not any longer feasible to perform a wider resection without evoking tumoral spread it was decided to continue with adjuvant radiation therapy and then closely follow up the patient in order to proceed with amputation only in case of recurrence. The other patient receives a scheduled clinical and imaging follow-up. Both patients continue to remain free of disease at the 24-month follow-up.

**Figure 6:**
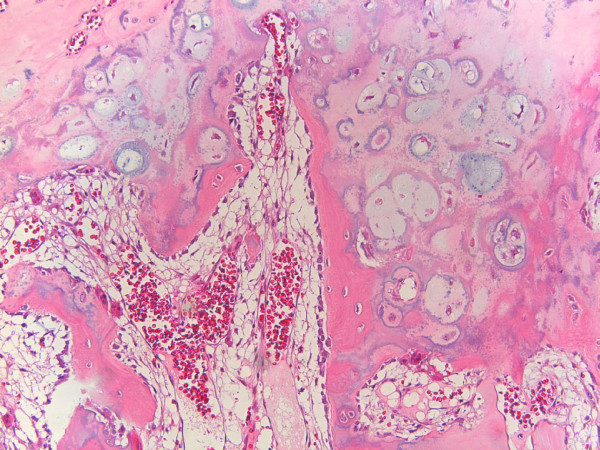
**Case 1 - The cartilaginous metaplasia seen in parosteal osteosarcoma can simulate the appearance of an osteochondroma due to the columnar arrangement of chondrocytes and the enchondral ossification (Hematoxiline&Eosine, 10 × magnification)**.

**Figure 7:**
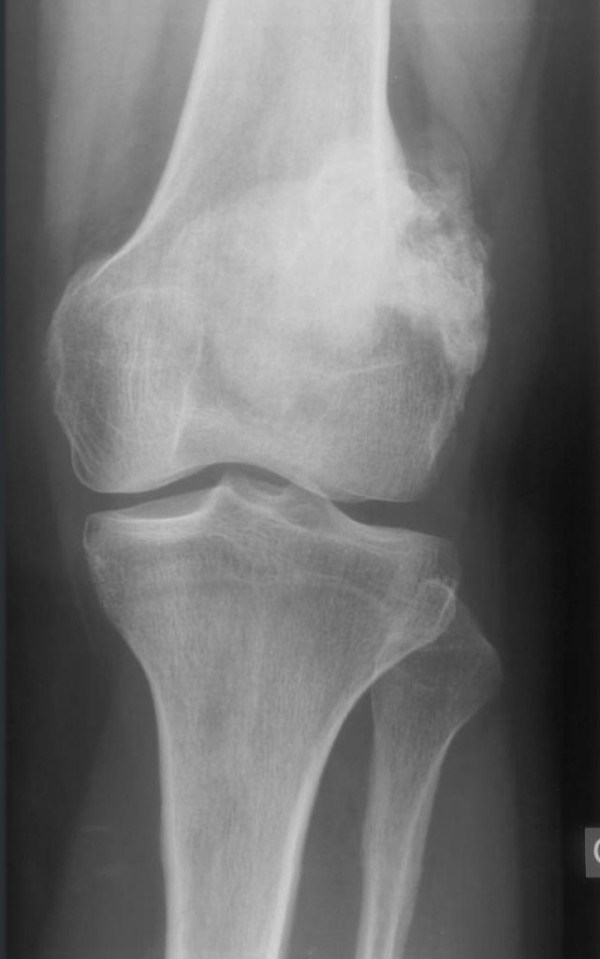
**Case 2 - AP view**.

**Figure 8:**
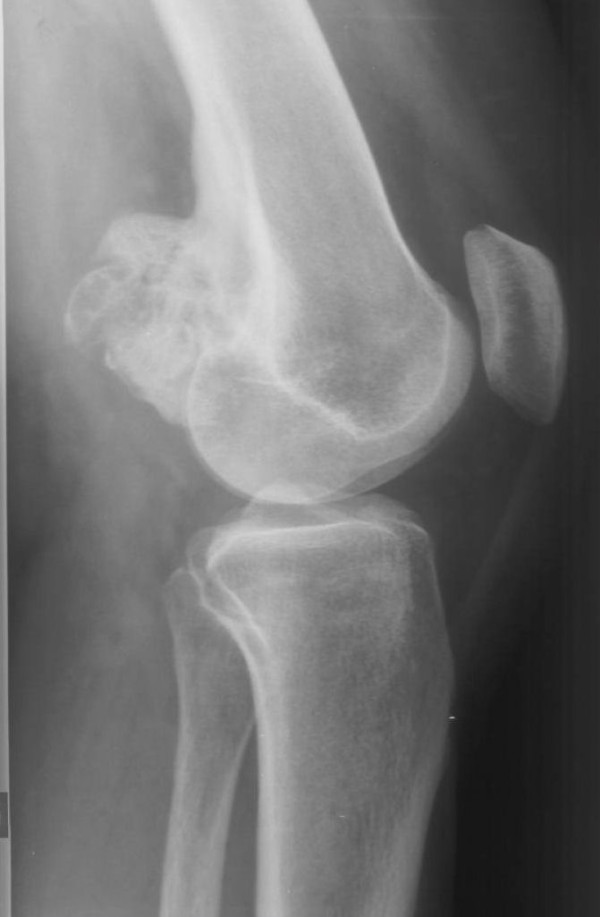
**Case 2 - lateral view: Radiographs exhibit a lobulated ossified mass attached with a broad base to the postero-lateral surface of the left distal femur with no radiolucent cleft**.

**Figure 9:**
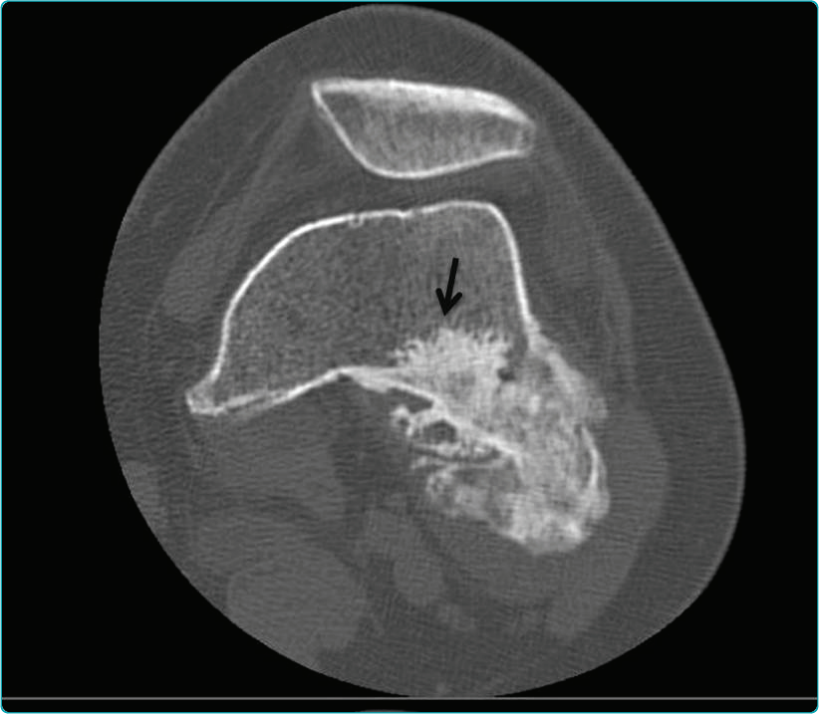
**Case 2 - Computed tomography demonstrates an intact and continuous cortex along with communication of the medullary cavities**. Limited invasion of the femoral medullar canal is also indicated (black arrow).

**Figure 10:**
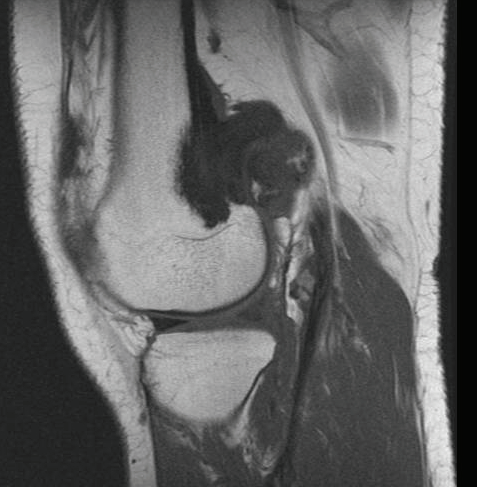
**Case 2 - T1WSE images**.

**Figure 11:**
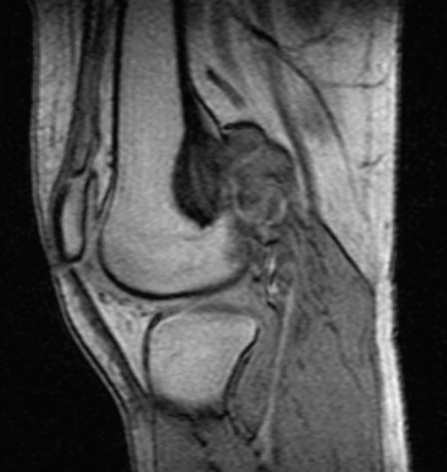
**Case 2 - post gadolinium images**. The mass has low SI on and displays moderate heterogeneous enhancement after contrast medium injection.

**Figure 12:**
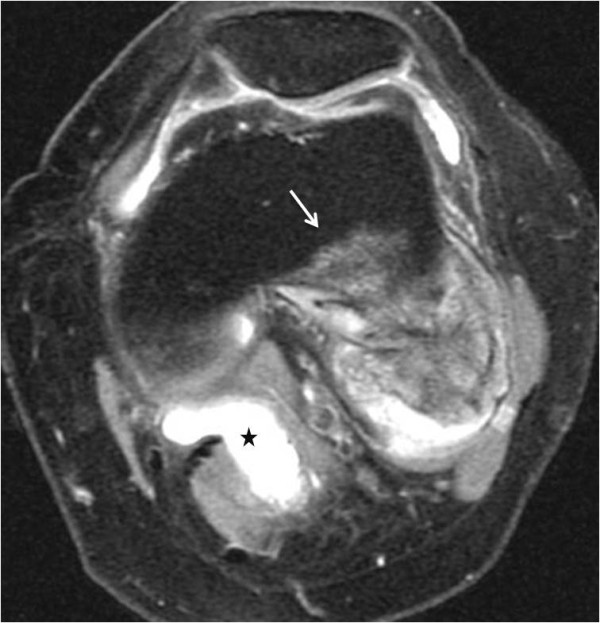
**T2 FAT SAT image shows a heterogeneous lesion containing hyperintense peripheral areas**. Medullar invasion is evident (white arrow). A coexisting popliteal cyst is also present (asterisk). Proposed drawing of the growth of the tumor, explaining the images.

## Discussion

POS is a rare bone-forming malignant surface tumor which carries a much better prognosis than central, high-grade osteosarcoma (approximately 90% of patients after 5 years) [2]. It constitutes about 1, 7% of all benign and malignant neoplasms of bone [6]. Originally it was described as "benign and malignant parosteal osteoma" by Geschickter and Copeland in 1951 [7]. Clinically it presents as a hard, immobile swelling with no or slight pain and frequently associated with loss of motion range of the neighboring joint [2,8]. The symptoms are often of prolonged duration [4]. Biologically, the tumor is slow-growing. Pulmonary metastases occur late in the course of the disease, usually following one or more local relapses. Therefore, surgical intervention focuses on the local control of the tumor [8]. Early recognition relies upon clinical suspicion and precise radiologic and pathologic evaluation. Apart from the posterior aspect of the distal femur, the tumor secondarily affects the proximal humerus followed by the proximal tibia [9]. Diaphyseal involvement is seen in up to 10% of cases [8]. In reference with the classic characteristics of the tumor, both masses were located at the postero-lateral portion of the distal femur and appeared hard, immobile and tender with associated limitation of the knee flexion on physical examination.

Macroscopically, POS presents as a dense and well-defined ossified mass attached to the underlying cortex [2]. On histologic grounds, the tumor mainly consists of hypocellular fibrous stroma with minimal atypia of spindle cells and extensive osteoid in the form of well-demarcated bony trabeculae, although smaller foci of cartilage are also encountered [1,2]. A cartilaginous component is observed in more than 50% of all POSs and in approximately 25% of cases this component lies at the periphery of the tumor. Pathologists and surgeons must recognize this cartilaginous component so as not to confuse POS with osteochondroma [4].

**Figure 13:**
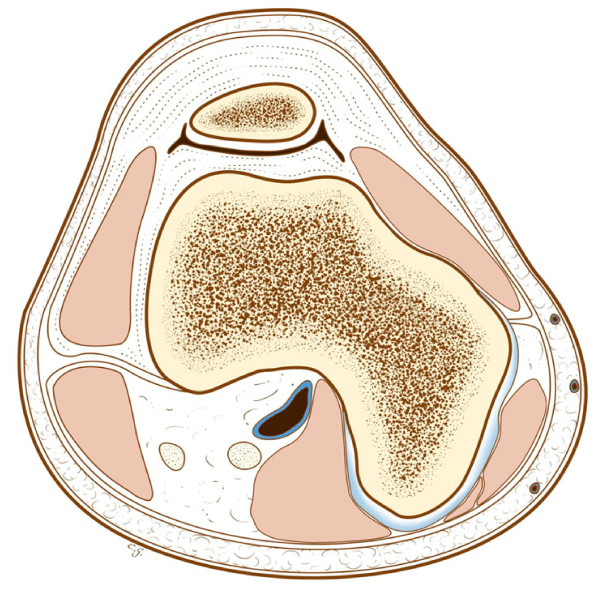
**What an osteochondroma in the same location would look like, with the cortex of the lesion in continuity with the cortex of the femur, normal marrow inside the lesion, and a cartilaginous cuff**.

**Figure 14:**
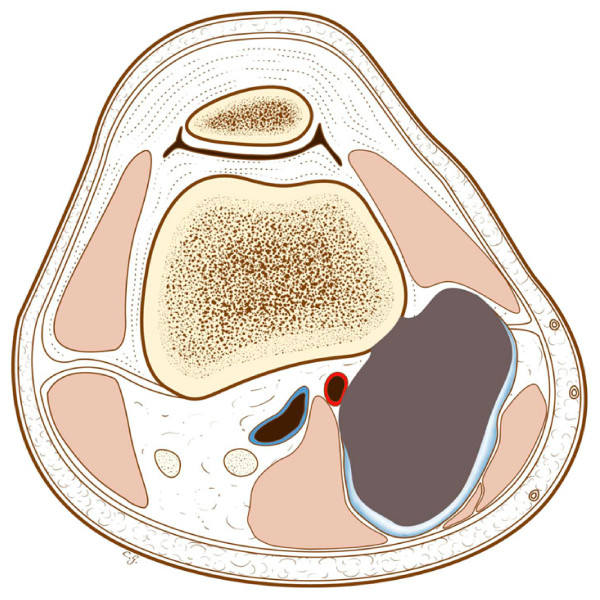
**The parosteal osteosarcoma at the beginning, with an almost intact cortex between the tumor and the normal bone**.

**Figure 15:**
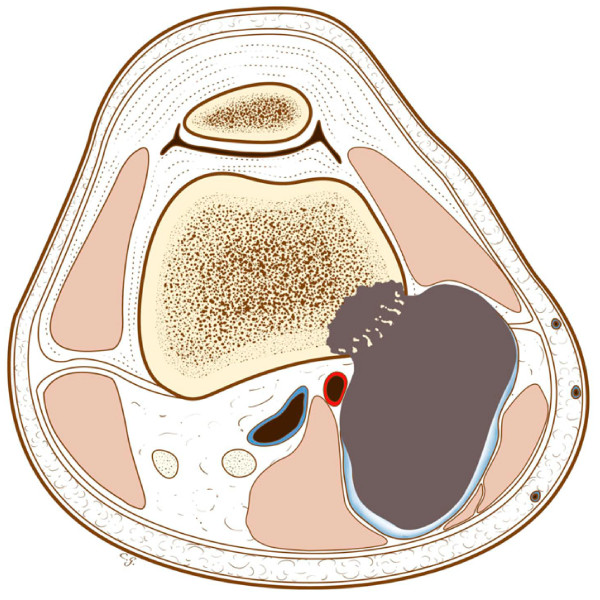
**One step forward, with invasion of the marrow**.

Radiographically, POS presents as a densely mineralized lobulated mass with irregular margins and attached to the subjacent cortex [2,9]. A characteristic finding is a linear radiolucent zone, separating the lesion from the host bone, except for the site of attachment, called the cleavage plane which represents the uncalcified thickened periosteum. However, this radiolucent cleft may be obliterated with advancing tumoral growth [3,9]. CT scans define accurately the extent of the tumor and cortical integrity [8]. MRI images vary in relation to tumors size as well as the presence of dense osteoid, cartilage, hemorrhage, necrosis or areas of high grade tumor or dedifferentiation. MRI is optimal for exhibiting the appropriate biopsy site and potential medullary invasion prior intervention [8]. Cortex continuity with some peripheral erosions is a useful diagnosis key. Conversely, cortex and medullary continuity are diagnostic features of osteochondroma. Perhaps the different appearances of the medullary cavities of the lesions compared to the host bone should have raised suspicion. Additionally a high grade osteosarcoma developed inside an osteochondroma is very rare, but possible and has a very similar pattern [10]. A peripheral cartilaginous cap may be visible on both parosteal osteosarcoma and osteochondroma. A biopsy made at the periphery of the lesion may reinforce the diagnosis of osteochondroma, as in one of our cases. The final correct diagnosis was established after resection, leading to inadequate margins. The disappearance of the cleavage plane and the preservation of a continuous femoral cortex encircling the tumors are probably attributed to the gradual destruction of the cortex by the slowly growing tumors (Figure 13, 14, 15, &16).

**Figure 16:**
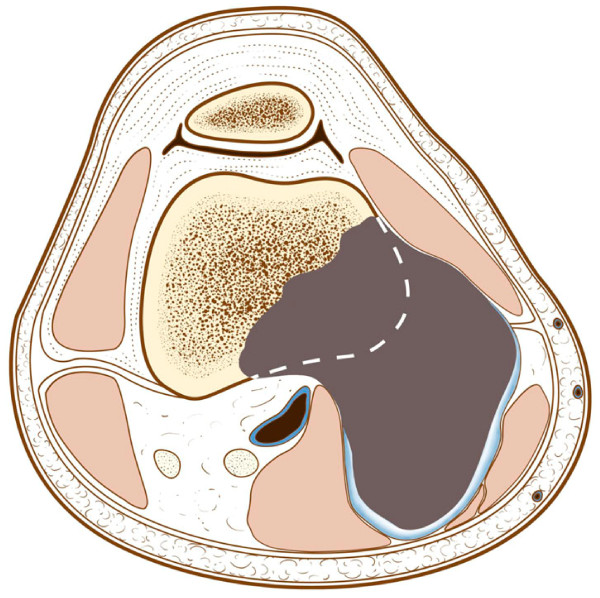
**The tumor has eroded and destroyed the cortex, that now looks in continuity with the cortex of the femur**.

The differential diagnosis of POS may also include other diverse entities such as mature juxtacortical myositis ossificans, parosteal osteoma, fracture callus, Nora's lesion, periosteal osteosarcoma and/or chondrosarcoma that can be easily distinguished [4,5,11]. In the study of Lin J et al [11], the term "osteochondromalike parosteal osteosarcomas" was used to describe six surface bone-forming malignancies characterized by cortical continuity and no evidence of medullary communication or invasion as shown in our cases.

In conclusion, osteochondroma is the main counterpart of POS in terms of imaging differential diagnosis. Cortex continuity and in a lesser degree merge of medullary cavities, which are by definition expected in osteochondromas, may be atypically seen in POS. Awareness of these features and thorough radiologic-pathologic correlation is crucial in avoiding erroneous diagnostic considerations and treatment.

## Competing interests

The authors declare that they have no competing interests.

## Authors' contributions

ZP wrote the article. MA and MG reviewed the histology. PT gave the second case, and checked the manuscript. GB checked the accuracy of the surgical part, CT reviewed the manuscript. DV proposed the subject and checked the review of the cases, references and global accuracy. All authors read and approved the final manuscript.

## References

[B1] Murphey M Robbin M Flemming D Temple T Kransdorf M From the Archives of the AFIP: The Many Faces of Osteosarcoma Radiographics 1997 17 1205 1231 9308111 10.1148/radiographics.17.5.9308111

[B2] Azura M Vanel D Alberghini M Picci P Staals E Mercuri M Parosteal osteosarcoma dedifferentiating into telangiectatic osteosarcoma: importance of lytic changes and fluid cavities at imaging Skeletal Radiol 2009 38 685 690 10.1007/s00256-009-0672-3 19271217

[B3] Jelinek J Murphey M Kransdorf M Shmookler B Malawer M Hur R Parosteal Osteosarcoma: Value of MR Imaging and CT in the Prediction of Histologlc Grade Radiology 1996 201 837 842 8939240 10.1148/radiology.201.3.8939240

[B4] Yong-Koo Nam Ryu Kyung Parosteal Osteosarcoma of the Scapula J Korean Med Sci 1999 14 586 88 10576159 10.3346/jkms.1999.14.5.586 PMC3054464

[B5] Subasi M Kapukaya A Buyukbayram H Bilici A Unusual benign bone lesion simulating parosteal osteosarcoma J Orthop Sci 2006 11 529 532 10.1007/s00776-006-1041-x 17013744

[B6] Lindell M Shirkhoda A Raymond KA Murray J HarIeT Parosteal Osteosarcoma: Radiologic-Pathologic Correlation with Emphasis on CT AJR 1987 148 323 328 3492111 10.2214/ajr.148.2.323

[B7] Geschickter CF Copeland MM Parosteal osteoma of bone: a new entity Ann Surg 1951 133 790 807 10.1097/00000658-195106000-00006 14838523 PMC1616939

[B8] Dönmez FY Tüzün Ü Başaran C Tunacĭ M Bilgiç B Acunaş G MRI findings in parosteal Osteosarcoma: correlation with histopathology Diagn Interv Radiol 2008 14 147 152 18814136

[B9] Suresha S Saifuddin A Radiological appearances of appendicular osteosarcoma: a comprehensive pictorial review Clin Radiol 2007 62 314 323 10.1016/j.crad.2006.11.002 17331824

[B10] Vanel D Picci P De Paolis M Mercuri M Osteosarcoma arising in an exostosis: CT and MRI imaging AJR 2001 176 259 260 11133584 10.2214/ajr.176.1.1760259

[B11] Lin J Yao L Mirra JM Bahk WJ Osteochondromalike parosteal osteosarcoma: A report of six cases of anew entity AJR 1998 170 1571 77 9609176 10.2214/ajr.170.6.9609176

